# Stages of Change Model for Participation in Physical Activity during Pregnancy

**DOI:** 10.1155/2013/193170

**Published:** 2013-02-04

**Authors:** Lene Annette Hagen Haakstad, Nanna Voldner, Kari Bø

**Affiliations:** ^1^Department of Sports Medicine, Norwegian School of Sports Sciences, P.O. Box 4014, Ullevål Stadion, 0806 Oslo, Norway; ^2^Department of Obstetrics and Gynecology, Oslo University Hospital, Rikshospitalet, 0424 Oslo, Norway

## Abstract

*Background*. The transtheoretical model (TTM) has been successful in promoting health behavioral change in the general population. However, there is a scant knowledge about physical activity in relation to the TTM during pregnancy. Hence, the aims of the present study were (1) to assess readiness to become or stay physically active according to the TTM and (2) to compare background and health variables across the TTM. *Methods*. Healthy pregnant women (*n* = 467) were allocated to the study from Oslo University Hospital, Norway. The participants filled in a validated self-administered questionnaire, physical activity pregnancy questionnaire (PAPQ) in gestation, weeks 32–36. The questionnaire contained 53 questions with one particular question addressing the TTM and the five stages: (1) precontemplation stage, (2) contemplation stage, (3) preparation stage, (4) action stage, and (5) maintenance stage. *Results*. More than half of the participants (53%) were involved in regular exercise (stages 4-5); however, only six specified that they had recently started an exercise program (stage 4). About 33% reported engaging in some physical activity, but not regularly (stage 3). The results showed that receiving advice from health professionals to exercise during pregnancy increased the likeliness of being in stages 4-5, while higher age, multiparity, pregravid overweight, unhealthy eating habits, pelvic girdle pain, and urinary incontinence were more prevalent with low readiness to change exercise habits (stages 1–3). *Conclusion*. According to the TTM, more than half of the participants reported to be physically active. Moreover, most of the participants classified as inactive showed a high motivational readiness or intention to increase their physical activity level. Hence, pregnancy may be a window of opportunity for the establishment of long-term physical activity habits.

## 1. Introduction 

To date, intervention studies show that exercise during pregnancy may enhance quality of  life and wellbeing, improve self-image and fitness, prevent excessive maternal weight gain, low back pain, pelvic girdle pain, and urinary incontinence, as well as decrease the risk of depression during pregnancy and postpartum [1–9]. Some observational studies have also reported associations between regular exercise during pregnancy and gestational diabetes, preeclampsia, shorter labor, fewer birth complications, and caesarean sections [10–17].

Present recommendations for exercise during pregnancy suggest that, in the absence of medical and obstetric complications, pregnant women should aim to perform at least 30 min of daily moderate intensity physical activity [18, 19]. However, studies have shown that most pregnant women do not exercise on a regular basis [20, 21], and that only 5–20% follow current exercise guidelines [22, 23]. Hence, pregnant women may have a great potential to increase physical activity and reduce the risk of inactivity related complications and illnesses. 

Antenatal care is part of public health promotion and prevention programs in most western countries, with pregnant women advised to attend between 5–8 visits throughout pregnancy [24]. Backe [25] found that the Norwegian antenatal healthcare system reaches almost 100% of pregnant women. Consequently, health care providers are in a unique position to inform and encourage pregnant women to start or continue specific and general exercise programs. Such programs may also help to establish long-term PA habits. 

In several settings, the transtheoretical model (TTM) of change has been successful in promoting behavioral change [26, 27]. According to this model, a specific health behavior develops over time and progresses through five stages which may be used to examine readiness to become and stay physically active (1) precontemplation, (2) contemplation, (3) preparation, (4) action, and (5) maintenance [26, 27]. However, there is a scant knowledge about the stages of change towards physical activity among pregnant women, and search on PubMed revealed no studies on this topic.

Hence, the specific aims of the present study were to (1) assess perceptions regarding readiness to become or stay physically active using the TTM and (2) to compare background and health variables across the five stages of the TTM among Norwegian pregnant women. 

## 2. Materials and Methods

### 2.1. Study Design and Participants

This study was part of a larger prospective study of determinants of macrosomic infants in Norway (STORK). Results from the main study have been published previously [16, 28]. Data collection to answer the present research questions was conducted through a self-administered questionnaire, (PAPQ) [29]. Healthy pregnant women were allocated to the study from the application form for birth at Oslo University Hospital between 2002 and 2005. Inclusion criteria were enrolment to the project before week 14–16 of gestation, having a singleton fetus, ability to answer PAPQ in gestation week 32–36, and being of Scandinavian origin. Exclusion criterion was pregestational diabetes or other serious diseases due to the primary aim of the main study. Of the 2145 women invited to participate in STORK, 678 accepted the invitation. However, 90 withdrew before inclusion. Fourteen women were excluded after routine ultrasound at gestation week 17-18, due to congenital disorders (*n* = 8) and twin births (*n* = 6). Further exclusions were two stillbirths, eleven relocations, and births at another hospital, and eight participants chose to withdraw. Consequently, 553 women were invited to participate in the present study. Of these, 467 (84.4%) filled in the PAPQ at home and returned the surveys at the last consultation with the midwife (NV), at mean pregnancy week 36.4 (SD = 1.7). Not all the participants answered every question, and therefore individual questions had varying response rates.

The STORK project followed the Helsinki declaration, and all the women gave written informed consent to participate. The Regional Committee for Medical and Health Research Ethics, Southern Norway, Oslo, and the Norwegian Social Sciences Data Services approved the project. 

### 2.2. Assessment Procedures and Outcome Measures

The PAPQ is a validated twelve-page questionnaire specifically designed to assess physical activity behavior in pregnant women [29] and includes information on background variables, health status and complaints, total physical activity level (commuting activities, occupational activities, housework, and family care activities), sedentary activities, recreational exercise, exercise motivation/barriers, and social support. More details of the questionnaire have been described elsewhere [30, 31]. 

The stages of change towards physical activity were assessed by a particular question aimed to classify the participants to one of five categories adapted from Godin and Shephard [32] and further developed by Prochaska et al. [33].  Table 1 shows the TTM scoring system, questionnaire categories, and motivational readiness to modify behavior. The participants were asked to pick the response category that most accurately described their current physical activity behavior or their interest for physical activity. Due to low response rate in the precontemplation stage and the action stage, and in agreement with a previous Norwegian study, the five stages were merged into two new variables in some supplementary statistical analyses [34]. Hence, participants in the precontemplation, contemplation, and preparation (stages 1–3) were classified as physically inactive (insufficiently PA), and participants in action and maintenance (stage 4-5) were classified as physically active (currently PA). Concurrent validity for TTM has been demonstrated with a significant association with the seven-day physical activity recall questionnaire [35], and the kappa index of test-retest intrareliability over a two-week period was 0.78 [35]. For the purpose of the present study, we used a translated Norwegian version, previously used in a study to assess motivational readiness to stay or increase physical activity level [34].

Maternal prepregnant weight was self-reported. Maternal weight gain was calculated as the difference between self-reported prepregnancy weight and the weight measured at the last clinical visit prior to delivery (pregnancy week 40.2, SD 1.3). The responsible midwife used a digital beam scale to measure the participant's body weight (kg). Classification of maternal weight gain and prepregnancy body mass index (BMI, kg/m^2^) was according to recommendations from the IOM: 12.7–18.2 kg weight gain for underweight women (prepregnancy BMI < 18.5), 11.4–15.9 kg weight gain for normal weight women (prepreg BMI of 18.5–24.9), 6.8–11.4 kg weight gain for overweight women (prepreg BMI of 25.0–29.9), and 5.0–9.1 kg weight gain for obese women (prepreg BMI ≥ 30) [36]. In the present study, 15 women had a prepregnancy BMI < 18.5, and 33 women had a prepregnancy BMI ≥ 30. These women were classified as either normal weight or overweight, and corresponding weight gain recommendations were used in the statistical analysis. It was presumed that more women in the precontemplation, contemplation, and preparatory groups would have less favorable weight gain compared to the action and maintenance groups. 

### 2.3. Statistical Analysis

All statistical analyses were conducted with SPSS statistical software version 18.0 for windows. Background variables are presented as frequencies, percentages, or means with standard deviations (SDs). The relationship between the TTM model and the selected variables, including health variables were assessed by one-way Anova, independent *t*-tests, or *χ* ^2^ as appropriate. In addition, we compared the TTM with present weight gain recommendations by the IOM [36]. The Spearman correlation coefficient was used to evaluate self-reported physical activity levels in the 3rd trimester (defined as vigorous leisure time physical activity ≥20 minutes once a week) and the TTM. Level of statistical significance was set at *P* < 0.05.

## 3. Results

Mean age of the participants was 31.6 years (range 20–49), mean prepregnancy BMI 23.6 (SD 3.7), and mean parity 1.3 (SD 0.5). The women were generally well educated, and 83% had education from college or university ≥4 years. The study group did not differ from nonparticipants giving birth at the same hospital, in mean maternal age, parity, gestational age at delivery, educational level, or marital status. Further information about background variables of the cohort has been presented elsewhere [30].

The distribution of participants within each stage of change is summarized in Table 2. According to the TTM, a large proportion of the participants reported to be somewhat or currently physically active, with 86.7% categorized in stages 3–5. Most participants were in maintenance (stage 5), followed by preparation (stage 3). Six women specified that they had recently started an exercise program (stage 4). 

As shown in Table 3, women defined in stages 1–3 (insufficiently PA) were somewhat older, multiparous, and pregravid overweight (BMI ≥ 25) and reported to be suffering from pelvic girdle pain and urinary incontinence. In total, 29.2% of the women defined their eating habits and nutritional status as unhealthy. A significantly higher proportion of these (64.7% versus 35.3%, *P* = 0.00) were categorized in stages 1–3 (insufficiently PA) as compared to stages 4-5, respectively. In contrast, more women (*n* = 167) of those having received advice to exercise during pregnancy were in the higher stages in the TTM (*P* = .001). No differences were found when comparing the stages of exercise with education, being sick listed in 3rd trimester, or daily smoking.

Since several cells in the crosstabs had expected count less than 5, caused by the low percentage of participants located in the precontemplation stage and action stage (Tables 3 and 4), we also performed additional tests analyzed with the five stages of change merged into two groups; insufficiently PA or currently PA. This did not change the overall results.

The majority of the participants (65.3%) had gained weight above the present recommendations from IOM. Comparing the participants and merging the five stages into two groups, we found that the difference between women in stages 1–3 (insufficiently PA) was not different in excessive weight gain compared to Current PA (stages 4-5) (72% versus 67%, *P* = 0.456). Mean maternal weight gain was nearly similar across the five groups, regardless of what stage of physical activity the women were located in (*P* = 0.558). 

## 4. Discussion

As far as we have ascertained, this is the first study to examine pregnant women's motivation for physical activity according to the TTM. In addition, several demographic and health indicators among women in the different stages of physical activity were compared. More than half of the participants were in stages 4-5, categorized as regularly active according to the TTM (Table 1). More than 32% were in stage 3, preparation. Only, 1.3% of the participants reported to be in stage 1 and had no intention to modify their physical activity behavior. Women being older, having children, suffering from pelvic girdle pain, reporting urinary incontinence, unhealthy eating habits, pregravid BMI ≥ 25, and not receiving advice from health care providers on how to perform PA during pregnancy were more often located in stages 1–3 (insufficiently PA) versus 4-5 (currently PA). Our study suggests that pregnancy may be a good time to guide and encourage women to be more physically active, as most women categorized as “insufficiently PA” reported a high motivational intention to become or increase their physical activity level.

The strength of the present study is the high response rate among the women receiving the PAPQ questionnaire. In addition, the population in STORK was similar in marital status, educational level, mean maternal age, parity, gestational age at delivery, and the baby's birth weight as compared to nonparticipants giving birth at Oslo University Hospital. When compared to the general Scandinavian pregnant population giving birth at Ullevål University Hospital, another major hospital in Oslo, the STORK participants included more nonsmokers, but were otherwise similar. Hence, the survey participants in the present study may be considered to be fairly representative for an urban Norwegian population of Scandinavian origin [16, 28]. We used a validated form of the TTM, and the association between self-reported physical activity levels and the stages of change found in the present study, may provide some evidence for the concurrent validity of the measure. The PAPQ included a broad range of determinants, ranging from demographic characteristics to lifestyle habits (such as smoking and diet), pregnancy complaints, and social support, including the physician's role to influence on physical activity level during pregnancy. In addition, the same midwife (NV) completed all weighing of the participants and calculated total maternal weight gain, as well as was available to answer any questions at the time when the participants handed in the questionnaire. 

An obvious limitation of cross-sectional surveys is that the design precludes the establishment of causation between variables and that most data are self-reported. Also, it is only a snapshot of the situation and may be biased by socially desirable responses. From 2002 to 2005, a total of 2145 women were randomly invited to participate in the STORK project. Unfortunately, due to logistic limitations, not all eligible women were approached, and unfortunately only about one fourth of the women accepted the invitation. Hence, although the response rate of eligible women to our study may be considered high, the representativeness of the STORK study can be questioned. The TTM was originally developed to be used in promoting or stopping a certain behavior [32]. In the present study, the model was used as a measure of pregnant women's readiness to become or stay physically active, as it has been used in other study populations [34]. 

Several studies have shown a decline in physical activity level before and throughout the course of pregnancy, and that only 5–20% follow the present exercise guidelines [18, 21–23, 30, 37]. Hence, this is in contrast to the participants of the present study and how they perceive their physical activity level according to the TTM. We found that more than 50% of the pregnant women reported that they were currently regularly active. 

The high amount of exercisers in the present study may be due to the main objective of the primary study, including evaluation of nutritional intake and physical activity on fetal macrosomia. Hence, the women who chose to participate may have had more interest in general health compared to nonparticipants. In addition, most of the participants reported a high educational level. Statistics Norway's survey of the living conditions in 2008, found that those with a high level of education were more physically active than those with a low level of education. However, in the present study, no significant association (*P* = .191) was found between the participants' level of education and stages of exercise during pregnancy. These results differ from other studies, finding that educational attainment is a strong determinant of stage for physical activity [38, 39].

In our study, significantly more women receiving advice from health care providers on physical activity during pregnancy reported to be in the higher stages of the TTM. Hence, our finding highlights the importance of precise and updated information, based on the current ACOG guidelines, to be distributed by health care professionals to their pregnant clients. Only 37% of the participant in the present study reported to have received advice about exercise. Considering that most pregnant women visit their health care provider on a regular basis, this may be a window of opportunity for providing information of the benefits of regular exercise during pregnancy. Hence, midwives and physicians should be encouraged to promote physical activity in pregnancy. This is supported by several studies reporting that pregnant women tend to follow the advice of health care providers regarding maternal weight gain [40, 41].

Previous studies have found that being sedentary before the onset of pregnancy is a risk factor not to start exercising when pregnant [30, 38, 42]. Our results support these findings, confirming that women who are accustomed to exercising prior to pregnancy are more likely to maintain this habit and that those not physically active prepregnancy do not start during pregnancy. Hence, to achieve higher rates of exercise during pregnancy, health promotion programs should target the general female population in their childbearing years. 

According to the review of Gaston and Cramp [38], being nulliparous has been a consistent predictor of regular exercise. In this study, more than 60% of the women categorized in the currently PA group (stages 4-5) were nulliparous. Therefore, to increase the level of exercise among multiparous women, activities allowing for personal time management and flexibility in terms of place and type of activity should be stimulated. Moreover, initiating supervised group activities and social support in a safe setting with qualified instructors may aid compliance to an exercise program.

An increasing proportion of women are overweight or obese at the start of their pregnancy [43], and it is assumed that this group is less likely to adapt and maintain the recommended levels of physical activity [44]. We found that fewer women with a high prepregnancy BMI (≥25) reported to be regularly active during pregnancy. 

No group differences were found between women reporting to be insufficiently PA or currently PA with respect to mean maternal weight gain or weight gain above the IOM references. This may be because maternal weight gain has been found to be independent of exercise [36]. We have not been able to find other studies examining the same relationship. Then again, data from published studies yield conflicting results regarding the impact of physical activity to control maternal weight gain [45, 46], and it has been suggested that participating in a physical activity may improve pregnancy outcomes independent of weight changes, and that even a small increase in activity level has a positive effect on mother and fetus [47]. 

Among the participants in the present study, about 1/4 reported to have a problem with urinary incontinence, and the majority of those women did not exercise regularly according to the TTM. This condition has been found to be a barrier to participation in physical activity due to embarrassment, feeling of discomfort, and possible increased leakage [48]. It is strong evidence that pelvic floor muscle training can prevent and treat urinary continence, and this should be taken into account when designing exercise program for women in all age groups, with extra emphasis during pregnancy [49]. 

## 5. Conclusion

Receiving advice from health professionals to exercise during pregnancy increased the likeliness of being in the action and maintenance stages. Higher age, multiparty, pregravid overweight, pelvic girdle pain, and urinary incontinence were more prevalent with lower readiness to change exercise habits. There is a need for more research to evaluate whether a TTM-based intervention is useful in promoting physical activity during pregnancy.

## Figures and Tables

**Table 1:** The readiness for physical activity stages of change scale (TTM).

Stage	Questionnaire response categories	Motivational readiness for change
(1)	I am currently not physically active and do not intend to engage in physical activity in the next six months	Pre-Contemplation (PC) (Physically inactive, no intentions to change)
(2)	I am currently not physically active, but I am thinking about getting more physically active in the next six months	Contemplation (C) (Physically inactive, intentions to change)
(3)	I currently do some physical activity, but not regularly	Preparation (P) (Physically active, but not regularly)
(4)	I am currently physically active, but have only begun doing so within the last six months	Action (A) (Regularly active, but only recently)
(5)	I am currently physically active and have done so for more than six months	Maintenance (M) (Regularly active)

**Table 2:** Proportions of women classified across the stages of change (TTM) based on self-report (*n* = 460).

	Motivational readiness for change	*n *	%
Preaction (Inactive)	Pre-Contemplation (stage 1)	6	1.3
	Contemplation (stage 2)	55	11.8
	Preparation (stage 3)	152	32.5

Postaction (Active)	Action (stage 4)	6	1.3
	Maintenance (stage 5)	241	51.6

Missing		7	1.5

**Table 3:** Comparison of background and health variables between the five stages of change (TTM). Results are presented as means with standard deviation (SD), in addition to number and percentages. With the exception of age and prepreg BMI, all variables are yes/no responses. Missing data are reported for each outcome as there are different response rates for several variables.

	Stages of exercise						
	PC	C	P	A	M	Missing	*P* value
	Stage 1	Stage 2	Stage 3	Stage 4	Stage 5		
Age	36.6 (4.2)	30.9 (4.1)	31.4 (4.0)	29.3 (4.1)	31.8 (3.9)	8	0.009
College/university education	4(80%)	43 (78.2%)	129 (84.9%)	6 (100%)	198 (82.8%)	9	0.2
Multiparous	5 (100%)	33 (60%)	74 (48.7%)	1 (16.7%)	97 (40.2%)	7	0.005
Pelvic girdle pain	3 (60%)	40 (72.7%)	72 (48%)	3 (50%)	142 (59.2%)	10	0.037
Urinary incontinence	4 (80%)	19 (34.5%)	44 (28.9%)	0	49 (20.3%)	7	0.004
Sick-listed 3rd trimester	2 (40%)	0	59 (39.9%)	0	78 (32.9%)	20	0.5
Daily smokers 3rd trimester	0	1 (1.8%)	4 (2.6%)	0	7 (2.9%)	7	1.0
Prepreg BMI	24.4 (3.6)	22.9 (3.7)	24.7 (4.0)	23.7 (4.6)	23.0 (3.2)	9	0.001
Prepreg BMI ≥25	0	12 (21.8%)	64 (42.1%)	1 (16.7%)	52 (21.6%)	7	0.001
“I consider my eating habits unhealthy”	1 (20%)	24 (45.3%)	61 (40.4%)	1 (16.7%)	46 (19.2%)	11	0.000
“I have received advice about PA in present pregnancy”	3 (75%)	14 (25.9%)	40 (26.8%)	1 (16.7%)	109 (46.0%)	16	0.001

*PC: precontemplation, C: contemplation, P: preparation, A: action, and M: maintenance.

**Table 4:** The relationship between maternal weight gain parameters and the TTM. Results are presented as means with standard deviation (SD), in addition to number and percentages.

Maternal weight gain parameters	Stage of exercise					Missing	*P* value
	PC	C	P	A	M
Weight gain (kg)	12.4 (7.4)	14.0 (4.2)	14.2 (5.7)	11.8 (2.7)	13.8 (5.2)	29	0.558
Exceeding IOM recommendations 2009	3 (60%)	38 (70.4%)	103 (72.5%)	4 (66.7%)	152 (67.9%)	29	0.634

*PC: precontemplation, C: contemplation, P: preparation, A: action, and M: maintenance.
